# A general framework for the Quantum Zeno and anti-Zeno effects

**DOI:** 10.1038/srep29497

**Published:** 2016-07-13

**Authors:** Adam Zaman Chaudhry

**Affiliations:** 1 School of Science & Engineering, Lahore University of Management Sciences (LUMS), Opposite Sector U, D.H.A., Lahore 54792, Pakistan

## Abstract

Repeated measurements can slow down (the quantum Zeno effect) or speed up (the quantum anti-Zeno effect) the temporal evolution of a quantum system. In this paper, a general treatment of the quantum Zeno and anti-Zeno effects is presented which is valid for an arbitrary system-environment model in the weak system-environment coupling regime. It is shown that the effective lifetime of a quantum state that is subjected to repeated projective measurements depends on the overlap of the spectral density of the environment and a generalized ‘filter function’. This filter function depends on the system-environment Hamiltonian, the state of the environment, and the measurement being performed. Our general framework is then used to study explicitly the Zeno to anti-Zeno crossover behaviour for the spin-boson model where a single two-level system is coupled to a bosonic environment. It is possible to not only reproduce results for the usual population decay case as well as for the pure dephasing model, but to also study the regime where both decay and dephasing take place. These results are then extended to many two-level systems coupled collectively to the bosonic environment to further illustrate the importance of the correct evaluation of the effective decay rate.

It is well-known that if a quantum system is subjected to rapidly repeated measurements, the temporal evolution of the quantum system slows down^1^. This effect, known as the Quantum Zeno effect (QZE), has attracted widespread interest both theoretically and experimentally due to its relevance to the foundations of quantum mechanics as well as possible applications in quantum technologies^2^,^3^,^4^,^5^,^6^,^7^,^8^,^9^,^10^,^11^,^12^,^13^,^14^,^15^,^16^,^17^,^18^,^19^,^20^,^21^,^22^,^23^. Surprisingly, however, it has also been found that if the measurements are not rapid enough, the opposite effect can occur, that is, the measurements can actually accelerate quantum transitions. This effect has been appropriately dubbed the quantum anti-Zeno effect (QAZE)^24^,^25^,^26^. Since this discovery, both the QZE and QAZE have been studied in many different physical setups such as superconducting qubits^10^,^23^,^27^, nanomechanical oscillators^28^, Josephson junctions^29^, disordered spin systems^30^, and localized atomic systems^31^. Generally speaking, the focus has been to study the population decay of quantum systems, whereby the quantum system is prepared in an excited state, and the system is thereafter repeatedly checked to see if the system is still in the excited state or not^24^,^26^,^28^,^29^,^30^,^31^,^32^,^33^,^34^,^35^,^36^,^37^. It has then been shown that the decay rate depends on the overlap of the spectral density of the environment and a measurement-induced level width^24^. This overlap changes as the measurement rate changes, generally leading to a crossover from the Zeno regime with large measurement rates to the anti-Zeno regime with relatively smaller measurement rates.

With increasingly sophisticated quantum technologies, it becomes important to investigate what happens beyond such population decay models. For instance, we can envisage repeatedly preparing not simply the excited state, but rather a superposition state of the ground and excited states. Such a superposition state will undergo not only population decay but also dephasing^38^. To this end, the QZE and the QAZE have been studied for a pure dephasing model in ref. 39. It was found therein that there are considerable differences between this case and the population decay case. For example, for the pure dephasing model in the weak coupling regime, the lifetime of the quantum state depends on the overlap of the spectral density of the environment and a ‘filter function’ that is different from the usual sinc-squared function obtained for the population decay model. Moreover, by examining the collective dephasing of many two-level systems, it was found that multiple Zeno and anti-Zeno regimes can be found. At the same time, however, these results are limited in the sense that they are only applicable for exactly solvable pure dephasing models. Nevertheless, these differences motivate us to investigate the QZE and the QAZE for more arbitrary system-environment Hamiltonians and state preparations. To this end, in this work, we derive an expression for the effective lifetime of a quantum state, subjected to repeated projective measurements, that is valid for weak system-environment coupling strength. This effective lifetime depends on the overlap between the spectral density of the environment and a generalized ‘filter function’ that in turn depends on the state that is repeatedly prepared, the environment correlation function, the system Hamiltonian parameters, the measurement interval, and the system-environment coupling. This expression is general in the sense that no assumption is made beforehand about the actual form of the system-environment Hamiltonian or the state that is being repeatedly prepared. The effective decay rate of the quantum state can thereby be controlled by suitably engineering the spectral density of the environment^40^.

Once we have derived a general expression for the effective lifetime of a quantum state, our main task then is to actually evaluate the generalized filter function. To illustrate the application of our formalism, the task of evaluating this filter function is first performed for the usual population decay model to show that we reproduce the well-known sinc-squared filter function. Similarly, we produce results that are in agreement with the exactly solvable pure dephasing model in the weak-coupling regime. We then consider the QZE and QAZE for the general spin-boson model where generally both population decay and dephasing take place. In particular, we show that the filter function is different for the general case as compared with the pure dephasing and decay cases and must be carefully evaluated. We then calculate the effective lifetime of the quantum state, and thereby the Zeno to anti-Zeno crossover behaviour, to further illustrate the differences as compared to the pure dephasing and decay cases. We next consider a collection of two-level systems collectively coupled to an environment that causes both dephasing and decay. In this case, we find that the effective decay rate is amplified depending on the number of particles coupled to the environment, thereby making the use of the correct filter function even more important.

## Results

### The general expression for the effective decay rate

We start by considering a general system-environment Hamiltonian written in the form *H* = *H*_*S*_ + *H*_*B*_ + *V*, where *H*_*S*_ is the system Hamiltonian, *H*_*B*_ is the Hamiltonian of the environment, and *V* describes the system-environment interaction. At time *t* = 0, we prepare the initial system state 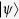. *N* repeated projective measurements, given by the projector 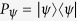, are now applied with equal time interval *τ*. It should be noted that before each measurement, we apply the operator 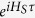 in order to remove the system evolution due to *H*_*S*_ itself^39^,^41^. In other words, following refs 39 and 41, we consider the so-called ‘dynamical fidelity’ to characterize the effective decay rate. If the survival probability of the quantum state is *S* after *N* time intervals, it is convenient to write *S* ≡ *e*^−Γ(*τ*)*Nτ*^, thereby allowing us to interpret 1/Γ(*τ*) as the effective lifetime of the quantum state. Alternatively, Γ(*τ*) can be interpreted as the effective decay rate of the quantum state. We assume that the system-environment interaction is weak enough such that we can safely ignore the build-up of the system-environment correlations as the system and the environment evolve together^39^. That is, we are neglecting any disturbance to the environment induced by measuring the system. In this case, *S* = [*s*(*τ*)]^*N*^ where *s*(*τ*) is the survival probability associated with one measurement. The effective decay rate is then 
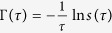
. Now, assuming that the coupling *V* can be written as *F* ⊗ *B* where *F* is an operator acting in the Hilbert space of the system and *B* is an operator acting in Hilbert space of the environment, we have found that the effective decay rate can be written as an overlap integral of the spectral density of the environment *J*(*ω*) and a generalized ‘filter function’ *Q*(*ω*, *τ*). That is,


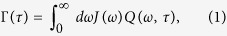


where the generalized filter function *Q*(*ω*, *τ*) is


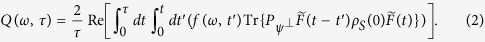


Here 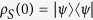, 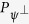 is a projector onto the subspace orthogonal to *ρ*_*S*_(0), and 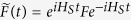. The environment correlation function is given by 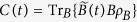 where 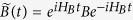, *ρ*_*B*_ is the initial state of the environment, and Tr_*B*_ denotes taking trace over the environment. The function *f*(*ω*, *t*) can then be extracted from 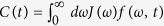. Details regarding the derivation of Eq. (1) can be found in the Methods section.

Thus, we have found that if the system-environment coupling is weak, the effective decay rate of a quantum state Γ(*τ*) depends on the overlap of the spectral density of the environment *J*(*ω*) and an effective filter function *Q*(*ω*, *τ*). This filter function depends on the frequency of the measurement, the state that is repeatedly prepared, the way that the system is coupled to the environment, and part of the environment correlation function. Note that the behaviour of Γ(*τ*) as a function of *τ* allows us to identify the Zeno and anti-Zeno regimes. One possible approach, followed for instance in refs 25 and 32, is to compare the ‘free’ effective decay rate in the absence of any measurements with the measurement-modified effective decay rate. An alternative approach, which we adopt in this paper, is to simply say that if Γ(*τ*) decreases when *τ* decreases, we are in the Zeno regime, while if Γ(*τ*) increases if *τ* decreases, then we are in the anti-Zeno regime^24^,^33^,^36^,^39^. We also note that for the more generalized system-environment coupling *V* = ∑_*μ*_*F*_*μ*_ ⊗ *B*_*μ*_, Γ(*τ*) is again given by Eq. (1), but now the filter function is


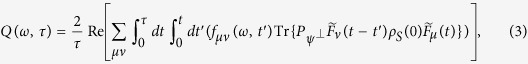


where *f*_*μν*_(*ω*, *t*) is extracted from 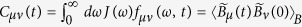.

### Application to the population decay model

To illustrate the formalism that we have developed, let us start from the system-environment Hamiltonian (we set *ħ* = 1 throughout this work)


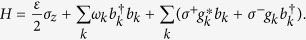


Here a two-level system with level spacing *ε* interacts with an environment, which is modeled as a collection of harmonic oscillators, and undergoes population decay. *σ*_*z*_ is the standard Pauli matrix, *σ*^+^ and *σ*^−^ are the raising and lowering operators respectively, *ω*_*k*_ are the frequencies of the environment oscillators, 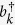 and *b*_*k*_ are the creation and annihilation operators for the oscillators, and the *g*_*k*_ describe the interaction strength between the two-level system and the environment modes. This system-environment Hamiltonian is widely used to study, for instance, spontaneous emission^42^. Note that this Hamiltonian is the same as


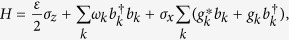


except that the non-rotating wave approximation terms have been dropped. To calculate the filter function, we identify *F*_1_ = *σ*^+^, 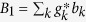, *F*_2_ = *σ*^−^ and 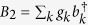. Let us first calculate the environment correlation functions. We find that 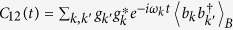, where 〈...〉_*B*_ denotes taking an average over the environment state. At zero temperature, all the oscillators are in their ground state, and we get 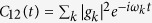. This sum is converted to an integral over the environment frequencies via the substitution 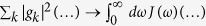, thus allowing us to identify *f*_12_(*ω*, *t*) = *e*^−*iωt*^. On the other hand, it is straightforward to show that *C*_11_(*t*) = *C*_22_(*t*) = 0, and *C*_21_(*t*) = 0 at zero temperature as well. The state initially prepared is the excited state which we denote by 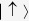. It follows that 
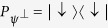
, where 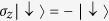. Using 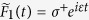 and 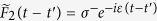, we find 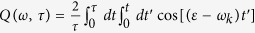. Performing the integrals, we end up with 
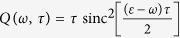
, which is the usual filter function^24^,^26^. Thus, our formalism reproduces the well-known sinc-squared function for the case where we study the Zeno to anti-Zeno transition in the context of population decay. In particular, if *τ* is small, then we obtain 
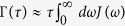
, thus putting us in the Zeno regime. However, for larger *τ*, decreasing *τ* can increase the overlap between *J*(*ω*) and *Q*(*ω*, *τ*), leading to the anti-Zeno effect.

### Application to the pure dephasing model

Let us now consider the system environment model specified by the Hamiltonian


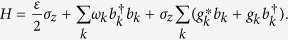


A two-level system with level spacing *ε* is interacting with an environment that is again modeled as a collection of harmonic oscillators. However, now there is no population decay. Instead, the system undergoes dephasing only, which is the reason why this model is known as the pure dephasing model^39^. Once again, we start by calculating the environment correlation function. Assuming the environment to be in the standard equilibrium state, that is 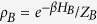 with *Z*_*B*_ the partition function, it is straightforward to show that 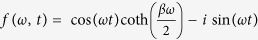. We consider the system state that we start off with to be 
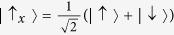
, where, as before, 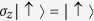 and 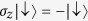. We repeatedly measure to check if the state is still 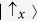 or not with time interval *τ*. Now 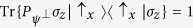, leading to 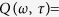 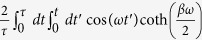. Performing the integrals and simplifying, we obtain 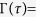

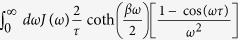
.

On the other hand, the pure dephasing model can be solved exactly. Using the exact solution, it can be shown that^39^ 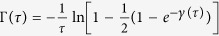, where 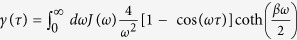. For weak coupling, however, 1 − *e*^−*γ*(*τ*)^ ≈ *γ*(*τ*), leading to 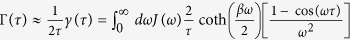, in agreement with the result obtained using the formalism that we have developed.

### Application with both dephasing and decay present

We now consider the more general system-environment model given by the Hamiltonian


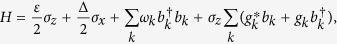


where Δ can be understood as the tunneling amplitude for the system, and the rest of the parameters are defined as before. This is the well-known spin-boson model^43^,^44^,^45^, which can be considered as an extension of the previous two cases in that we can now generally have both population decay and dephasing taking place. We revert to the usual dephasing model by setting Δ = 0, while setting *ε* = 0 leads to the population decay Hamiltonian (with the non-rotating wave approximation terms now included) after rotation about the *y*-axis. Experimentally, such a model can be realized, for instance, using superconducting qubits^23^,^46^,^47^ and the properties of the environment can be appropriately tuned as well^48^.

We start from the general system initial state 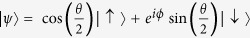, where the states 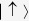 and 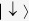 are defined as before, and *θ* and *ϕ* are parameters that characterize the state preparation. Measurements, with time interval *τ*, are now carried out to check if the system is still in this state or not. To evaluate *Q*(*ω*, *τ*), we first need to find the environment correlation function. We again consider the environment to be in a thermal equilibrium state. Consequently, as in the pure dephasing model, *f*(*ω*, *t*) = cos(*ωt*)coth(*βω*/2) − *i* sin(*ωt*). Next, we find 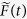. This is done using the standard commutation relation [*σ*_*k*_, *σ*_*l*_] = 2*iε*_*klm*_*σ*_*m*_. A straightforward application of the Baker-Hausdorff lemma^49^ shows that 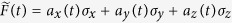, with


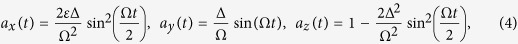


and Ω^2^ = *ε*^2^ + Δ^2^. Now, given 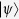, we can deduce that 
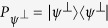
 with 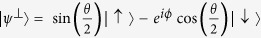. The rest of the calculation for the filter function proceeds in a straightforward manner, leading to


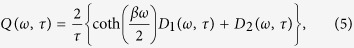


with 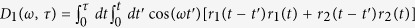, and 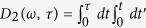 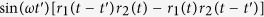. Here *r*_1_(*t*) = −*a*_*x*_(*t*)cos *ϕ* cos* θ* − *a*_*y*_(*t*)sin *ϕ* cos *θ* + *a*_*z*_(*t*)sin* θ*, and *r*_2_(*t*) = *a*_*y*_(*t*)cos* ϕ* − *a*_*x*_(*t*)sin *ϕ*.

We next evaluate *D*_1_(*ω*, *τ*) and *D*_2_(*ω*, *τ*). Although the integrals can be done, the final analytical results for arbitrary *θ* and *ϕ* are, unfortunately, very long and not very illuminating. Instead, let us choose *θ* = *π*/2 and *ϕ* = 0. This choice of state has the advantage that we can then compare our results with the already well-known cases of the population decay model and the pure dephasing model. In particular, the choice Δ = 0 then corresponds to the pure dephasing model (in the weak coupling limit). On the other hand, *ε* = 0 corresponds to (almost) the population decay model since we can rotate both the system-environment Hamiltonian and the state that we are measuring about the *y*-axis. The only difference is that we now have additional non-rotating wave approximation terms, but we expect these additional terms to not play a role in the weak system-environment coupling regime that we are in. It must be emphasized that we are now no longer restricted to these two models only. Rather, by varying the values of *θ*, *ϕ*, *ε*, and Δ, we can explore regimes where both dephasing and relaxation play a role in the quantum Zeno and anti-Zeno effects.

**Figure 1:**
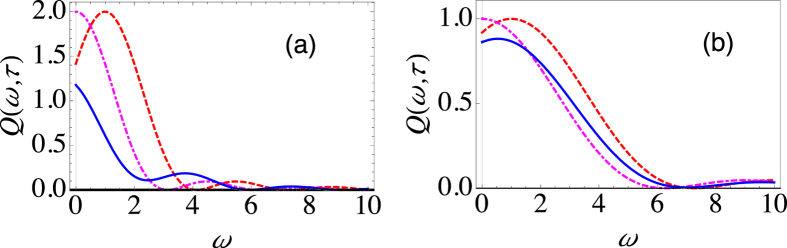
Behaviour of the filter function. (**a**) Graph of *Q*(*ω*, *τ*) (at zero temperature) as a function of *ω* for *τ* = 2 with Δ = 1 and *ε* = 0 (dashed, red curve), Δ = 0 and *ε* = 1 (dot-dashed, magenta curve), and Δ = 1 and *ε* = 2 (solid, blue curve). Here, we have set *θ* = *π*/2 and *ϕ* = 0. Throughout, we use dimensionless units with *ħ* = 1. (**b**) Same as (**a**), except that now *τ* = 1.

With *θ* = *π*/2 and *ϕ* = 0, the calculation for *D*_1_(*ω*, *τ*) and *D*_2_(*ω*, *τ*) becomes less laborious since *r*_1_(*t*) and *r*_2_(*t*) simplify greatly. Analytical expressions for *D*_1_(*ω*, *τ*) and *D*_2_(*ω*, *τ*) are given in the Supplemental Material. A few points are in order. First, if Δ = 0, then *D*_2_(*ω*, *τ*) = 0, while *D*_1_(*ω*, *τ*) = [1 − cos(*ωτ*)]/*ω*^2^, which, as expected, leads back to the filter function for the pure dephasing model. Similarly, for zero temperature with *ε* = 0, *D*_1_(*ω*, *τ*) and *D*_2_(*ω*, *τ*) simplify such that we get back the filter function for the population decay model. For intermediate values of *ε* and Δ, however, the filter function is very different. This is illustrated in Fig. 1 where *Q*(*ω*, *τ*) has been plotted (in dimensionless units with *ħ* = 1) as a function of *ω* for two different values of *τ*. The dashed, red curve is the filter function for the population decay model with *ε* = 0 and Δ = 1, and is thus consequently peaked at *ω* = 1 in both Fig. 1(a,b). The dot-dashed, magenta curve is the filter function for the pure dephasing model (with *ε* = 1 and Δ = 0), while the solid, blue curve shows the intermediate case with *ε* = 2 and Δ = 1. Clearly, if the value of *τ* is not too small, the filter function for the intermediate case resembles neither the population decay model nor the pure dephasing model. This means that for an arbitrary spectral density of the environment, the value of Γ(*τ*) is expected to be quite different with both Δ and *ε* non-zero as compared to the population decay and pure dephasing cases. On the other hand, as *τ* becomes smaller, we must have that Γ(*τ*) → 0, and thus the filter functions must start to resemble each other more for smaller *τ*. This is entirely consistent with what we see in Fig. 1(b).

**Figure 2:**
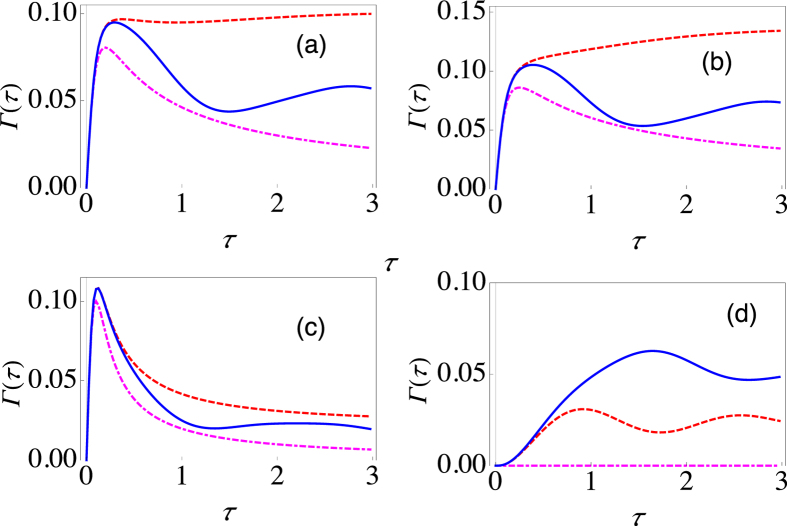
Behaviour of the effective decay rate for the spin-boson model. (**a**) Graph of Γ(*τ*) (at zero temperature) as a function of *τ* for *θ* = *π*/2 and *ϕ* = 0 with Δ = 2 and *ε* = 0 (dashed, red curve), Δ = 0 and *ε* = 2 (dot-dashed, magenta curve), and Δ = 2 and *ε* = 2 (solid, blue curve) for an Ohmic environment (*s* = 1). We have set *G* = 0.01 and *ω*_*c*_ = 10. (**b**) Same as (**a**), except that now we have a sub-Ohmic environment with *s* = 0.8. For large *τ*, the dashed, red curve becomes horizontal with Γ(*τ*) approximately equal to 0.14. (**c**) Same as (**a**), except that now we have a super-Ohmic environment with *s* = 2. (**d**) Same as (**a**), except that now we have *θ* = 0 and *ϕ* = 0.

With the different behaviour of the filter function, we expect that when there is both dephasing and decay, the effective decay rate will be quite different compared to either of the aforementioned cases, and thus the Zeno and anti-Zeno behaviour is expected to be considerably modified. Let us now explicitly examine this claim for the case of sub-Ohmic, Ohmic and super-Ohmic environments. To this end, we introduce the spectral density as 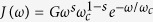. Here the parameter *s* characterizes the Ohmicity of the environment. Namely, *s* = 1 corresponds to an Ohmic environment, *s* > 1 gives a super-Ohmic environment, while *s* < 1 corresponds to a sub-Ohmic environment. *G* is a dimensionless parameter characterizing the system-environment coupling strength, and we have introduced an exponential cutoff function with a cutoff frequency *ω*_*c*_. Let us first investigate an Ohmic environment. In Fig. 2(a), we illustrate the behaviour of the effective decay rate Γ(*τ*) as a function of the measurement interval *τ*, clearly showing the very different behaviour of Γ(*τ*) when both population decay and dephasing are present. For the population decay case (the dashed, red curve), with the chosen values of the system-environment parameters, Γ(*τ*) by and large decreases as *τ* decreases. Thus, we are in the Zeno regime. On the other hand, the pure dephasing case (dot-dashed, magenta curve) displays a distinct Zeno regime and an anti-Zeno regime. For small values of *τ*, Γ(*τ*) decreases as *τ* is decreased, meaning that a shorter measurement interval helps to protect the quantum state, thus putting us in the Zeno regime. For larger values of *τ*, however, as the *τ* is decreased the opposite happens, namely, Γ(*τ*) increases as *τ* decreases, thus indicating the anti-Zeno regime. Now, when both dephasing and population decay take place (solid, blue curve), the behaviour of Γ(*τ*) is considerably different. Besides the quantitative differences in the values of Γ(*τ*), the effective decay rate displays qualitatively different behaviour in the sense that we now have clearly distinct multiple Zeno and anti-Zeno regions. This is evident from the fact that Γ(*τ*) displays multiple extrema, meaning that sometimes decreasing the measurement interval reduces the decay rate, while sometimes the opposite happens.

The effective decay rate, as we have emphasized, depends on the overlap of the spectral density and the generalized filter function. Thus, we expect that changing the environment, and in particular the Ohmicity parameter, should alter the decay rate, at least quantitatively. In Fig. 2(b), we have calculated the decay rate with a similar set of parameters as was done with the Ohmic environment. The only difference is that we are now considering a sub-Ohmic environment with *s* = 0.8. Once again, while the population decay case exhibits the QZE, and the pure dephasing case exhibits both the QZE and the QAZE, the more general case displays multiple transitions between the QZE and the QAZE. We have also examined a super-Ohmic environment [see Fig. 2(c)] with *s* = 2. Now the population decay case also exhibits both the QZE and the QAZE. With both dephasing and population decay present, we again have multiple Zeno to anti-Zeno transitions, but these transitions are less clear cut as compared to what we observed in the previous cases. These results illustrate the importance of the type of environment in determing the QZE-QAZE crossover behaviour.

Before moving on, let us also investigate the Zeno and anti-Zeno effects for a different state preparation. Namely, we choose *θ* = 0 and *ϕ* = 0. Once again we find *D*_1_(*ω*, *τ*) and *D*_2_(*ω*, *τ*) and use these to find the effective decay rate. Exact expressions for *D*_1_(*ω*, *τ*) and *D*_2_(*ω*, *τ*) can be found in a similar way as before (refer to the Supplemental Material). With these expressions in hand, we can investigate the behaviour of Γ(*τ*). In Fig. 2(d), we have shown the behaviour of Γ(*τ*) for the population decay Hamiltonian (dashed, red curve), the pure dephasing case (dot-dashed, magenta curve), and the intermediate case (solid, blue curve) with an Ohmic environment. For pure dephasing, Γ(*τ*) remains zero. This makes sense since the state that we are repeatedly preparing, namely 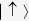, does not evolve under the action of the pure dephasing Hamiltonian. On the other hand, the population decay Hamiltonian leads to both decay and dephasing, since, after rotation about the *y*-axis, the state that is repeatedly prepared is a superposition of the 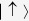 and 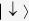 states. Although both the population decay Hamiltonian and the intermediate case display multiple Zeno to anti-Zeno transitions, the actual value of the decay rate, in general, is considerably different for the two cases. These two cases also differ in the values of *τ* for which the transitions take place. By now, it should be clear that using the usual simple sinc-squared filter function to analyze a system undergoing both dephasing and decay would be incorrect.

### Application to the large spin-boson model

**Figure 3:**
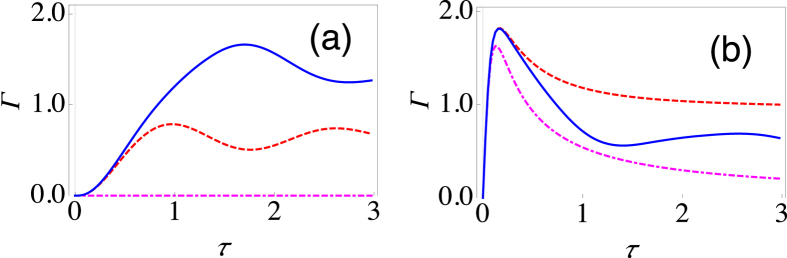
Effective decay rate for the large spin-boson model. (**a**) Behaviour of Γ(*τ*) (at zero temperature) as a function of *τ* for the initial state 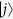 with Δ = 2 and *ε* = 0 (dashed, red curve), Δ = 0 and *ε* = 2 (dot-dashed, magenta curve), and Δ = 2 and *ε* = 2 (solid, blue curve) for a sub-Ohmic environment (*s* = 0.8). We have set *G* = 0.01 and *ω*_*c*_ = 10, and the number of particles is taken to be 20. (**b**) Same as (**a**), except that now the state that we are repeatedly preparing is 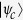 and the environment is super-Ohmic with *s* = 1.5.

Let us now consider *N*_*s*_ two-level systems interacting collectively with a common environment. The system-environment Hamiltonian is now


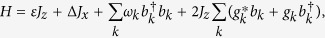


where *ε* and Δ are the level spacing and tunneling amplitude respectively for each two-level system, and *J*_*x*_ and *J*_*z*_ are the standard angular momentum operators. This Hamiltonian can be considered to be a generalization of the usual spin-boson model to a large spin *j* = *N*_*s*_/2^39^,^50^,^51^. Physical realizations include a two-component Bose-Einstein condensate^52^,^53^ that interacts with a thermal reservoir via collisions^51^. Once again, we assume that the system-environment coupling is weak so that our formalism applies. The initial state is chosen to be 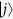 such that 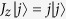, and we repeatedly check, with time interval *τ*, whether the system state is still 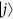 or not. We note that we can easily deal with some other choice of initial state as well. As before, our task is to calculate *Q*(*ω*, *τ*), the details of which are given in the Methods section. The important point to note here is that the decay rate is amplified depending on the number of two-level systems coupled to the environment. Namely, we find that the filter function is now


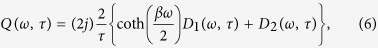


with 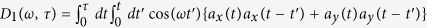, and 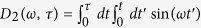

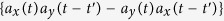
. The filter function *Q*(*ω*, *τ*) for this case is then *N*_*s*_ times the filter function for a single spin-half particle with *θ* = 0 and *ϕ* = 0. In other words, Γ(*τ*) for *N*_*s*_ particles is simply *N*_*s*_ times the decay rate for a single particle. For illustration purposes, we have plotted Γ(*τ*) as a function of the measurement interval *τ* in Fig. 3(a) for *N*_*s*_ = 20 and a sub-Ohmic environment. It should be noted that the amplification of Γ(*τ*) now makes the use of the correct filter function even more critical. For instance, for the population decay Hamiltonian in Fig. 3(a) with Δ = 2 and *ε* = 0, the survival probability after five measurements with time interval *τ* = 1 is approximately 0.02, while the survival probability for the more general Hamiltonian with Δ = 2 and *ε* = 2 after five measurements with the same time interval *τ* = 1 is ten times smaller.

Suppose now that we prepare the state of each two-level system in a coherent superposition such that the total state is 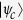 where 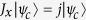. This time we obtain


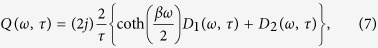


with 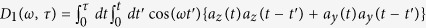, and 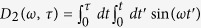 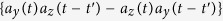. We again find that Γ(*τ*) for *N*_*s*_ particles coupled collectively to the environment is given by *N*_*s*_ times the decay rate for a single particle. Quantitative results are presented in Fig. 3(b) for *N*_*s*_ = 20 with a super-Ohmic environment (*s* = 1.5). Again, due to the amplification of the effective decay rate, it becomes very important to use the correct filter function.

## Discussion

We have derived an expression for the effective decay rate of a quantum state in the presence of repeated measurements which is valid when the system-environment coupling is weak. This expression implies that the effective decay rate of the quantum state depends on the overlap of the spectral density of the environment and a generalized filter function. We have shown that our formalism for calculating the effective decay rate reproduces the well-known results for the population decay model and the pure dephasing model. Thereafter, we demonstrated that our formalism allows us to study the Zeno and anti-Zeno effects for the spin-boson model in a rigorous fashion under the assumption that the system-environment coupling strength is weak. We have found qualitative and quantitative differences in the behaviour of the decay rate as a function of the measurement interval when both decay and dephasing are present as compared to the relatively simpler population decay and pure dephasing models. Finally, by considering many two-level systems coupled collectively to a common environment, we have observed that the decay rate is amplified depending on the number of two-level systems. Consequently, it is even more crucial to use the correct filter function to evaluate the effective decay rate. We should also emphasize that our framework can be used to study other system-environment models as well. Experimental implementations of the ideas presented in this paper are expected to be important for measurement-based quantum control, as well as stimulating further work on the Quantum Zeno and anti-Zeno effects.

## Methods

### Derivation of the effective decay rate

The system-environment Hamiltonian is *H* = *H*_*S*_ + *H*_*B*_ + *V*, where we recall that *H*_*S*_ is the system Hamiltonian, *H*_*B*_ is the Hamiltonian of the environment, and *V* describes the system-environment interaction. At time *t* = 0, we prepare the initial system state 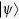. To calculate the effective decay rate, we find the survival probability *s*(*τ*), provided that we start from the pure system state 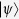. In order to do so, let us first find an expression for *ρ*_*S*_(*τ*), the state of the system at time *t* = *τ*, just before the system is measured. Now, the total system-environment state at this time is *ρ*_tot_(*τ*) = *U*(*τ*)*ρ*_tot_(0)*U*^†^(*τ*), where *U*(*τ*) represents the unitary time-evolution due to the total system-environment Hamiltonian *H*. We can then find the state of the system at time *τ* as *ρ*_*S*_(*τ*) = Tr_*B*_{*U*(*τ*)*ρ*_tot_(0)*U*^†^(*τ*)}, where Tr_*B*_ denotes taking partial trace over the environment. Generally speaking, however, it is usually impossible to find *U*(*τ*) exactly. Instead, we resort to perturbation theory, which can be used if we assume that the system-environment interaction is weak. We write *U*(*τ*) as *U*(*τ*) = *U*_0_(*τ*)*U*_*I*_(*τ*), where 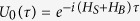 is the ‘free’ unitary time-evolution operator, and *U*_*I*_(*τ*) can be expanded as a perturbation series, that is, *U*_*I*_(*τ*) = 1 + *A*_1_ + *A*_2_ + …, where *A*_1_ and *A*_2_ are the first and second order corrections respectively. The system density matrix at time *τ* is then


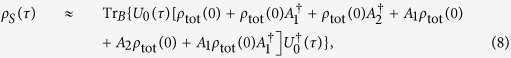


correct to second order in the system-environment coupling strength.

To proceed further, we assume that the initial system-environment state can be represented as *ρ*_tot_ = *ρ*_*S*_(0) ⊗ *ρ*_*B*_, that is, the initial system-environment state is a simple product state. This is a rather standard assumption in the open quantum systems literature, but it should be noted that the validity of this assumption is questionable when the system-environment coupling strength is strong and/or the number of particles collectively coupled with the environment is large^54^,^55^,^56^. Moreover, we also assume that we can write *V* = *F* ⊗ *B*, where *F*(*B*) is an operator belonging to the system (environment) Hilbert space. The more general case where *V* = ∑_*μ*_*F*_*μ*_ ⊗ *B*_*μ*_ can be dealt with via a straightforward extension. The operators *A*_1_ and *A*_2_ intro-duced above are then found to be 
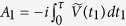
, and 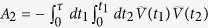, with 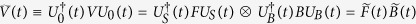, where 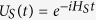 and 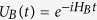. We now simplify Eq. (8) term by term. First, we find that 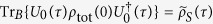, where 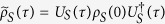 is the system density matrix if the system and environment are not coupled together. Next,


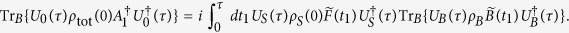


But 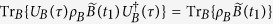, which is zero for the system-environment models usually considered^45^. Similarly, 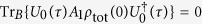. The next term is


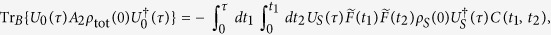


with the environment correlation function *C*(*t*_1_, *t*_2_) defined as 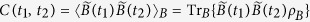. Similarly,


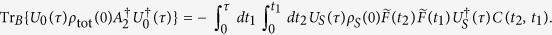


Finally, 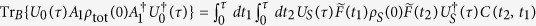. Using the fact that 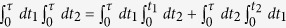, we can manipulate further to obtain


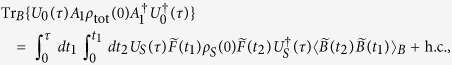


where h.c. denotes hermitian conjugate. Putting all the terms back together, the system density matrix can be written as


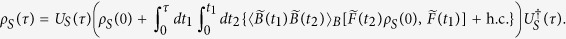


We can simplify this further by noting that the environment correlation function 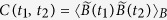 generally depends on the time difference *t*_1_ − *t*_2_ only. This motivates us to introduce *t*′ = *t*_1_ − *t*_2_. The system density matrix at time *τ* then becomes


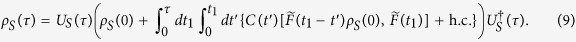


with the simplified notation 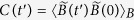 for the environment correlation function.

Once we have the expression for the density matrix at time *τ*, we can compute the survival probability. This survival probability can be simply calculated as one minus the probability of getting some result other than the state 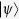 that we have started off with. Consequently, it is useful to define the projection operator 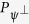 that projects onto the subspace orthogonal to the state 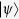. Moreover, we must also keep in mind that, just before performing the measurement, we perform a unitary operator (which is implemented on a very short time-scale) in order to remove the evolution due to the system Hamiltonian itself. This unitary operator then removes the *U*_*S*_(*τ*) and 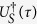 that can be found to the left and right of the right hand side of Eq. (9). Thus, we can write the survival probability as


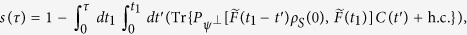


where Tr denotes simply taking the trace over the system only, and we have used the fact that 
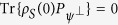
. Using the fact that for any operator *X*, Tr(*X* + *X*^†^) = 2Re[Tr(*X*)], where Re denotes taking the real part, we further simplify to


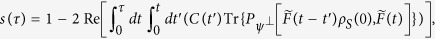


and we have replaced *t*_1_ with *t* for notational simplicity. Since 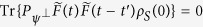, we get


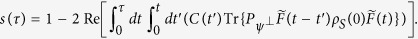


Now, the environment correlation function will typically be of the form 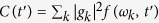, where *g*_*k*_ is the coupling strength of the system with mode *k* of the environment, and *f*(*ω*_*k*_, *t*′) is simply a function containing the remaining information about *C*(*t*′). This sum is usually replaced by an integral over the frequencies of the environment via the substitution 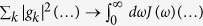, thereby introducing the spectral density *J*(*ω*) of the environment. Also, what we are really interested in is the effective decay rate 
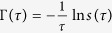
. For weak coupling strength, the deviation of *s*(*τ*) from unity is small. Therefore, the effective decay rate can be approximated as 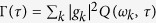, where


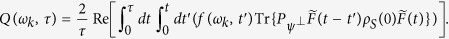


By introducing the spectral density, we can write instead 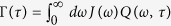, where the generalized ‘filter function’ *Q*(*ω*, *τ*) is


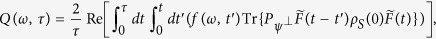


thereby ending up with Eq. (1). The approach given above can easily be generalized to a coupling of the form 
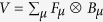
, leading to Eq. (3).

### Finding the effective decay rate for the large spin-boson model

We first consider the case where we repeatedly prepare the state 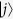 given by 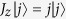. We begin by calculating 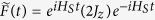. Using the standard commutation relations, [*J*_*k*_, *J*_*l*_] = *iε*_*klm*_*J*_*m*_, and the Baker-Hausdorff lemma, we find that


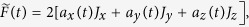


with *a*_*x*_(*t*), *a*_*y*_(*t*) and *a*_*z*_(*t*) as defined in Eq. (4). Now, 
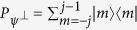
, leading to


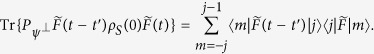


By observing that 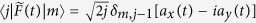 and simplifying, we find the filter function given in Eq. (6).

Let us now calculate the filter function for the case when we repeatedly prepare the state 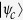 given by 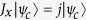. To investigate the Zeno and anti-Zeno effects now, it is best to transform to a rotated frame, that is, we rotate both the system-environment Hamiltonian and the state that we are repeatedly preparing. In other words, we use the fact that


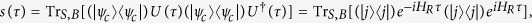


where the transformed Hamiltonian is


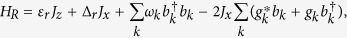


with *ε*_*r*_ = Δ and Δ_*r*_ = −*ε*. We then find that 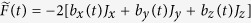, with 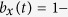 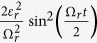, 
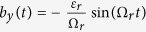
, and 
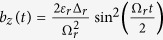
, where 
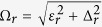
. The rest of the calculation proceeds in a very similar way to what we did with the state 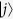 and leads to


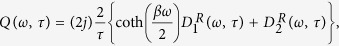


with 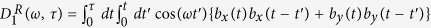, and 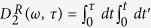 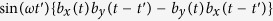. Now, it is easy to show that *b*_*x*_(*t*) = *a*_*z*_(*t*) and *b*_*y*_(*t*) = −*a*_*y*_(*t*). Thus, we obtain the filter function given in Eq. (7).

## Additional Information

**How to cite this article**: Chaudhry, A. Z. A general framework for the Quantum Zeno and anti-Zeno effects. *Sci. Rep.* **6**, 29497; doi: 10.1038/srep29497 (2016).

## Supplementary Material

Supplementary Information
