# Supplementary material for: A general framework for the Quantum Zeno and anti-Zeno effects
Source: Sci Rep. 2016 Jul 13;6:29497. doi: 10.1038/srep29497 (PMC4942788; doi:10.1038/srep29497)
Supplement: Supplementary Information [file srep29497-s1.pdf]

Supplemental Material: A general framework for the Quantum Zeno and anti-Zeno effects

Adam Zaman Chaudhry[1,*]

[1] School of Science & Engineering, Lahore University of Management Sciences (LUMS), Opposite Sector U, D.H.A, Lahore 54792, Pakistan

In this Supplemental Material, we use the same notation as introduced in our main text. We present the analytical expressions for $D_1(\omega,\tau)$ and $D_2(\omega,\tau)$ for a single two-level system. For $\theta=\pi/2$ and $\phi=0$, we find that $D_1(\omega,\tau)=X_1/X_2$, and $D_2(\omega,\tau)=X_3/X_4$, where

$$\begin{aligned}
X_1 =& 3\Delta^4\omega^4 + 4\Omega^2\cos(\omega\tau)\left[\varepsilon^2(\varepsilon^2-\omega^2)(\omega^2-\Omega^2) - \Delta^2\omega^2\left(\Delta^2+\omega^2\right)\cos(\Omega\tau)\right] + \\
& \Delta^2\omega\left[4\Omega^3\left(\varepsilon^2-2\omega^2\right)\sin(\omega\tau)\sin(\Omega\tau) - \omega\varepsilon^2(\omega^2-\Omega^2)(\cos(2\Omega\tau)-4\cos(\Omega\tau))\right] - \\
& 8\Omega^6\left(\Delta^2+\omega^2\right) - 3\Delta^2\omega^2\Omega^2\left(\Delta^2+\omega^2\right) + \Omega^4\left(4\Delta^4+15\Delta^2\omega^2+4\omega^4\right) + 4\Omega^8, \\
X_2 =& 4\Omega^4\omega^2\left(\omega^2-\Omega^2\right)^2, \\
X_3 =& \Delta\Big\{\omega\Omega(\varepsilon^2-\Delta^2-\omega^2)\sin(\omega\tau)\sin(\Omega\tau) + \cos(\omega\tau)[\varepsilon^2(\Omega^2-\omega^2) - (\Omega^2\left(\Delta^2+\omega^2\right)+\Delta^2\omega^2-\Omega^4)\cos(\Omega\tau)] + \\
& \Omega^2(\Delta^2+\omega^2) + \Delta^2\omega^2 + \varepsilon^2(\omega^2-\Omega^2)\cos(\Omega\tau) - \Omega^4\Big\}, \\
X_4 =& \omega\Omega^2\left(\omega^2-\Omega^2\right)^2.
\end{aligned}$$

On the other hand, for $\theta=0$ and $\phi=0$, we find that

$$\begin{aligned}
X_1 =& \Delta^2\Big\{\omega^2\Omega^2\left(\omega^2+3\Omega^2\right) - 4\omega\Omega^3\left(\omega^2+\varepsilon^2\right)\sin(\omega\tau)\sin(\Omega\tau) + 4\Omega^2\cos(\omega\tau)\left[\varepsilon^2(\omega^2-\Omega^2) - \omega^2\left(\Omega^2+\varepsilon^2\right)\cos(\Omega\tau)\right] + \\
& \omega^2(\Omega^2-\omega^2)\left[\Delta^2\cos(2\Omega\tau)+4\varepsilon^2\cos(\Omega\tau)\right] + \varepsilon^2\left(3\omega^4-3\omega^2\Omega^2+4\Omega^4\right)\Big\}, \\
X_2 =& 4\Omega^4\omega^2\left(\omega^2-\Omega^2\right)^2, \\
X_3 =& 4\Delta^2\varepsilon\left\{\omega\cos\left(\frac{\omega\tau}{2}\right)\sin\left(\frac{\Omega\tau}{2}\right) - \Omega\sin\left(\frac{\omega\tau}{2}\right)\cos\left(\frac{\Omega\tau}{2}\right)\right\}^2, \\
X_4 =& \omega\Omega^2\left(\omega^2-\Omega^2\right)^2.
\end{aligned}$$

*Electronic address: adam.zaman@lums.edu.pk
